# Type of aortic valve replacement influences ascending aortic flow characteristics - a pilot study using 4D flow MRI

**DOI:** 10.1186/1532-429X-15-S1-P245

**Published:** 2013-01-30

**Authors:** Florian von Knobelsdorff-Brenkenhoff, Ralf F Trauzeddel, Alex J Barker, Henriette Gruettner, Michael Markl, Jeanette Schulz-Menger

**Affiliations:** 1 Working Group Cardiovascular MRI, Experimental and Clinical Research Center (Charite Medical Faculty and MDC) and HELIOS Clinics Berlin, Berlin, Germany; 2 Department of Radiology, Feinberg School of Medicine, Northwestern University, Chicago, IL, USA; 3 Department of Biomedical Engineering, McCormick School of Engineering, Northwestern University, Chicago, IL, USA

## Background

Prosthesis-related alterations of blood flow in the ascending aorta after aortic valve replacement (AVR) may influence aortic remodeling. The study aimed at analyzing ascending aortic flow characteristics after various types of AVR.

## Methods

Flow-sensitive four-dimensional magnetic resonance imaging (4D-flow) was acquired in 38 AVR patients (n=9 mechanical, n=8 stentless bioprosthesis, n=14 stented bioprosthesis, n=7 autograft) and 9 healthy controls. Analysis included grading of vortex and helix flow (0-3 point scale), assessment of systolic flow eccentricity (1-3 point scale), and quantification of the segmental distribution of peak systolic wall shear stress (WSS_peak_) in the ascending aorta.

## Results

**Figure 1:**
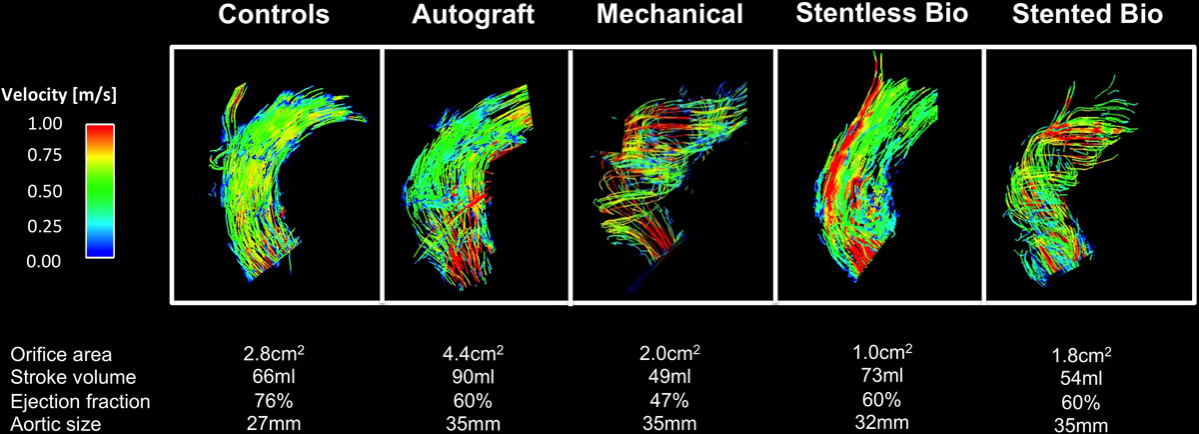
Visualization of the blood flow in the ascending aorta using particle traces during peak systole: Each image is selected to be representative for the specific valve group.

**Figure 2:**
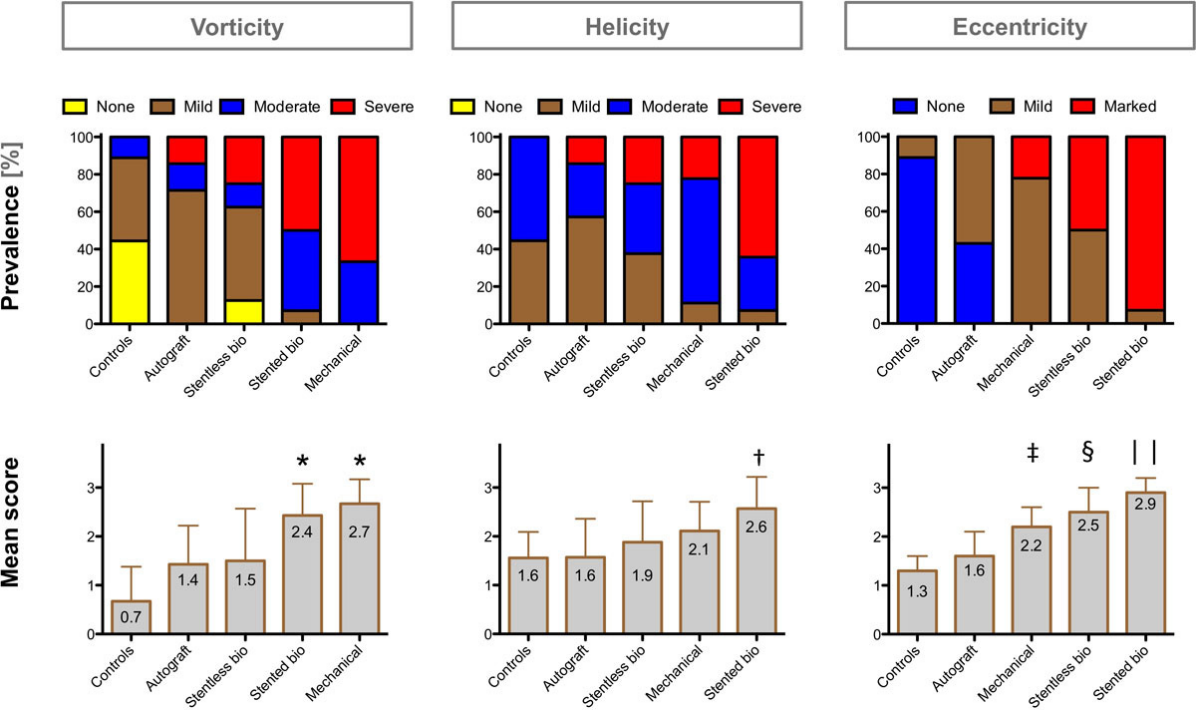
Evaluation of vorticity, helicity and eccentricity of blood flow: The upper row shows the frequency of each score for the various AVR groups and controls. The lower row depicts the mean ± SD scoring results. Please note that the order of the groups varies between the three columns. (* p<0.05 vs. stentless, autografts and controls; † p<0.05 vs. stentless, autografts and controls; ‡ <0.05 vs. controls; $ p<0.05 vs. stented, autografts, controls; | |p<0.05 vs. stentless, mechanical, autografts and controls)

Compared to controls, mechanical prostheses showed the most distinct vorticity (2.7±0.5 vs. 0.7±0.7; p<0.001), while stented bioprostheses exhibited most distinct helicity (2.6±0.7 vs. 1.6±0.5; p=0.002) (Figures 1 and 2). Instead of a physiologic central flow, all stented, stentless and mechanical prostheses showed eccentric flow jets mainly directed towards the right-anterior aortic wall. Stented and stentless prostheses showed an asymmetric distribution of WSS_peak_ along the aortic circumference, with significantly increased local WSS_peak_ where the flow jet impinged on the aortic wall. Local WSS_peak_ was higher in stented (1.4±0.7N/m^2^) and stentless (1.3±0.7N/m^2^) compared to autografts (0.6±0.2N/m^2^; p=0.005 and p=0.008) and controls (0.7±0.1N/m^2^; p=0.017 and p=0.027). Autografts exhibited lower absolute WSS_peak_ than controls (0.4±0.1N/m^2^ vs. 0.7±0.2N/m^2^; p=0.003).

## Conclusions

The flow characteristics in the ascending aorta in all AVR types were different from volunteers with native aortic valve, and they differed between the various types of AVR.

## Funding

FvKB is supported by the Else Kröner-Fresenius Stiftung (Bad Homburg, Germany). AJB is funded by the Whitaker Postdoctoral and Fulbright Grants (New York, USA). MM is supported by the NMH Excellence in Academic Medicine (EAM) Program 'Advanced Cardiovascular MRI Research Center' (Chicago, USA).

